# Health systems strengthening for noncommunicable disease control and healthy aging: integrated actions in Aruba and Curaçao

**DOI:** 10.26633/RPSP.2019.55

**Published:** 2019-06-07

**Authors:** Malhi Cho, Melissa Marchand, Enrique Vega, Reynaldo Holder, Silvana Luciani, Jeanine Constansia-Kook, José Moya

**Affiliations:** 1 Regional Office of the World Health Organization (WHO) for the Americas Regional Office of the World Health Organization (WHO) for the Americas Pan American Health Organization (PAHO) Washington, DC United States Pan American Health Organization (PAHO), Regional Office of the World Health Organization (WHO) for the Americas, Washington, DC, United States.; 2 PAHO consultants PAHO consultants Washington, DC United States PAHO consultants, Washington, DC, United States.; 3 Ministry of Health, Environment, and Nature Ministry of Health, Environment, and Nature Willemstad Curaçao Ministry of Health, Environment, and Nature, Willemstad, Curaçao.; 4 PAHO Country Office for the Bolivarian Republic of Venezuela and the Netherland Antilles PAHO Country Office for the Bolivarian Republic of Venezuela and the Netherland Antilles Caracas Venezuela PAHO Country Office for the Bolivarian Republic of Venezuela and the Netherland Antilles, Caracas, Venezuela.

**Keywords:** Health systems, aging, noncommunicable diseases, Aruba, Curaçao., Sistemas de salud, envejecimiento, enfermedades no transmisibles, Aruba, Curaçao, Sistemas de saúde, envelhecimento, doenças não transmissíveis, Aruba, Curaçao

## Abstract

Caribbean countries are experiencing social, epidemiological, and demographic transitions shaped by the growing elderly population and the rise of noncommunicable diseases (NCDs)—now responsible for 78% of all deaths. These circumstances demand rethinking the model of care to improve health outcomes and build more sustainable health systems with new orientations in policy, service delivery, organization, training, technology, and financing. Policy must be aimed towards healthy living, leveraging interventions that ensure healthy aging. The health system must proactively structure interventions to reduce the incidence of new NCD cases and to prevent related complications. Interventions should be focused on optimizing the individual’s capacity, functional ability, and autonomy within adapted environments, as well as with the necessary preventive, long-term care, self-care, community care, and health system support.

## RESUMEN

Los países del Caribe están experimentando transiciones sociales, epidemiológicas y demográficas como resultado del envejecimiento de la población y el aumento de la prevalencia de las enfermedades no transmisibles (ENT), que en la actualidad causan el 78% de todas las defunciones. Estas circunstancias exigen replantear el modelo de atención para mejorar los resultados de salud y establecer sistemas de salud más sostenibles mediante nuevos enfoques en políticas, prestación de servicios, organización, capacitación, tecnología y financiamiento. Las políticas deben tener como propósito procurar una vida saludable, aprovechando las intervenciones que garanticen el envejecimiento saludable. El sistema de salud debe ser proactivo, estructurando las intervenciones para reducir la incidencia de nuevos casos de ENT y para prevenir las complicaciones relacionadas. Las intervenciones deben centrarse en optimizar la capacidad funcional, la autonomía y el desenvolvimiento general de la persona dentro de un entorno adaptado y con el apoyo necesario del sistema de salud en materia de atención preventiva, cuidados a largo plazo, de autocuidado y de atención en la comunidad.

## RESUMO

Os países do Caribe estão passando por transições sociais, epidemiológicas e demográficas configuradas pela população de idosos cada vez maior e a ascensão das doenças não transmissíveis (DNTs), já responsáveis por 78% de todas as mortes. Essas circunstâncias exigem repensar o modelo de atenção para melhorar os desfechos de saúde e construir sistemas de saúde mais sustentáveis, com novas orientações de política, prestação de serviços, organização, treinamento, tecnologia e financiamento. As políticas devem ser orientadas para a vida saudável, alavancando intervenções que asseguram o envelhecimento saudável. O sistema de saúde deve estruturar proativamente intervenções para reduzir a incidência de novos casos de DNT e a prevenir as complicações relacionadas. As intervenções devem se concentrar na otimização da capacidade do indivíduo, das habilidades funcionais e da autonomia dentro de ambientes adaptados, e também nas ações preventivas a para assistência de longa duração, no autocuidado, na atenção na comunidade e no apoio pelos sistemas de saúde.

## BACKGROUND AND CONTEXT

Two main factors impacting the sustainability of Caribbean health care systems are the increasing cost of health care and the growing elderly population. These must be carefully weighed to determine the most effective and efficient mix of hospital, primary health, and community-based care to address the needs of the population and to promote healthy aging. Higher life expectancy in the Caribbean has resulted in a concomitant increase in the population 60 years of age and older, particularly over 80 years. In fact, the fastest growing population group in the Americas is over 80 and this group is expected to triple globally by 2050 (1 – 3).

**FIGURE 1:**
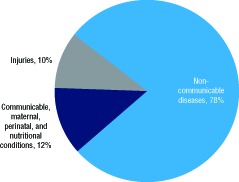
Causes of death: English Caribbean

***Source***: Prepared by the authors with data from the Regional Mortality Database, Pan American Health Organization, 2017.

While extended life expectancy is largely related to successful public health interventions, it has not been accompanied by improved health; instead, people are living longer with disease and disability. In the English Caribbean, noncommunicable diseases (NCDs) account for 78% of total deaths (Figure 1), 38% of which occur prematurely (4). NCDs also lead to an increased prevalence of disability, which requires additional health planning and policy.

For Aruba and Curaçao, the demographic shift means that by 2030 approximately 28% of the population will be 60 years of age and over (5). NCDs, primarily cardiovascular disease and cancer, are currently responsible for 79% of deaths in Curaçao and 85% in Aruba (4). The prevalence of NCDs is expected to rise as the population continues to age, as health systems provide earlier detection, and as improved treatments extend life expectancy—people will live more years with disease and will require more health care.

Risk factors for NCDs—notably tobacco use, harmful use of alcohol, unhealthy diet, and physical inactivity—already affect a high percentage of the population of the Dutch Caribbean, mainly overweight and obesity. In Curaçao, 65% of the population is overweight or obese, 20% has hypertension, 9% has diabetes, and 15.5% uses tobacco (6). While a complete dataset is unavailable for Aruba, recent data indicate a similar trend, with 77% of the population being overweight (82.8% of men and 72.5% of women) and 40.8% considered obese (7, 8).

Most health concerns begin in early life, making a healthy life style essential to reduce chronic disease risk over the life course (9). As populations age, the complexity of health conditions increases, requiring robust coordination of care to address increased disability and frailty and multiple chronic conditions. Furthermore, since most people over 80 depend on others for their care, aging impacts the social and health structures far beyond this age group.

The current model of care in Aruba and Curaçao is a biomedical model focused mainly on treatment provided in hospitals as episodic and curative care. Most funding is currently directed toward treating diseases and their complications. Conversely, preventive care and promotion of healthy lifestyles, which could delay or reduce complications from NCDs—the number one cause of death in these countries—remains underfunded.

The countries of the Dutch Caribbean requested that PAHO convene a platform for discussion to explore alternatives for tackling future health care challenges. However, due to a hurricane, only the representatives of Aruba and Curaçao were able to attend.

The workshop of experts and representatives of health authorities from Aruba and Curaçao was convened on 9 – 11 November 2017 in Curaçao to discuss actions for facing health care challenges related to the aging population. Participants represented the health policy departments and health care providers of Aruba and Curaçao, and included PAHO advisors on NCDs and Healthy Life Course and Health Systems and Services. This document presents the workshop’s discussions on challenges, affordability, and sustainability of health systems in Aruba and Curaçao, and the experts’ recommendations for effectively addressing the issues.

## WORKSHOP DISCUSSION

The health authorities and health workers from Aruba and Curaçao expressed concerns regarding the affordability and sustainability of the current health care model as it faces the burden of an elderly population with chronic conditions. The participants represented the health policy departments and health care providers of Aruba and Curaçao and PAHO advisors on NCDs, Healthy Life Course and Health Systems and Services.

The increased cost of health care for the aging population must be carefully weighed to determine the most effective and efficient mix of the various levels of health services and community-based health care necessary for promoting healthy aging. The workshop findings outline a path to reorient the health systems of Aruba and Curaçao by improving and adjusting models of care by:

Strengthening primary health care, transitioning to people-centered and integrated care, and facilitating self-management of care;

Prioritizing healthy aging with the life course approach and making necessary adjustments for providing affordable quality of care to the growing elderly population; and

Promoting healthy public policies and healthy lifestyles through the life course approach for reducing risks for NCDs.

### Integrated approach

The workshop discussion also focused on the need for an integrated approach between health and other sectors and strengthening community-based actions, tailored to the needs of the population. Integration of health system and services can avoid conflicting medical advice and over-prescribing and is especially important for the complex issues of the aging population and the population affected by chronic disease, multiple conditions, and disability. Integrated and comprehensive services are pivotal to improving the quality of care, optimizing finances, better management of human and other resources, and providing mechanisms for ensuring treatment compliance. These actions can be further supported by designating a care coordinator or care team and designing care plans.

**Social determinants of health**

Workshop discussions also addressed the role of social determinants of health in improving outcomes. Health services can provide top technology and high levels of service, but if a person returns to the same conditions that caused the illness in the first place, health care efforts have been mostly ineffective. Policy decision is an important process to address social determinants of health, using the legal framework and the capacities of the health authority to contribute to healthy, safe, and sanitary environments.

### Community-based initiatives

Community health spans many different types of organizations and programs, from social organizations, self-care groups, and spaces for information exchange and learning, to community-based initiatives, such as green spaces, community and school gardens, and healthy cooking. Environments must be adapted for the elderly population, accounting for aspects such as mobility, vision, and hearing impairments, and focusing on maintaining the functional autonomy of this population.

Policies must also strengthen the ability of individuals and communities to take an active role in health issues, providing tools for self-management and getting the community engaged, committed, and empowered in health care decisions. Community-based health also has an important role to play in overcoming the challenges of an aging population, supporting environments that create autonomy for older individuals with disabilities, chronic diseases, or multi-morbidity. This applies not only to public spaces, but also to personal ones where national policies on importation and installation of mobility equipment may impact one’s ability to adapt the surroundings to support sustained functional ability.

### Human resources for health

With regards to human resources for health, it is necessary for health workers to have adequate profiles, skills, and knowledge. Varied levels of training and the needs of caregivers must be considered when designing community programs to support the aging and disabled. The health system and community must jointly prepare for a shift in the demographics of caregivers to effectively organize actions and interventions, and develop a system that will meet the future needs of the entire health care team.

### Self-management

Individuals are constantly faced with decisions concerning their health, though they may be in contact with the health care system only a few hours a year. So, 99% of the time, decisions are made individually, outside of the health system (2) and without consulting a doctor. These decisions comprise a multitude of behaviors, from eating and exercise habits, to seeking preventive services, taking medication, and so on.

The workshop discussed different strategies, tools, and methodologies that might encourage people, including the elderly, to become active participants in their health, ensuring that their day-to-day decisions support healthy living and healthy aging. Health systems and social services must work together to support people with declining or diminished functional capacity (10, 11), encouraging independence in daily living activities within their own environment.

By designing and offering robust self-management tools adapted to an individual’s customs and habits, a health system can teach individuals how to optimally manage their own health on a day-to-day basis. Self-management involves a group of tasks that an individual should undertake to live well with one or more chronic conditions. It elevates the importance of the individual’s role as the main agent responsible for caring for one’s own health. Self-management tools focus on providing knowledge, skills, motivation, and confidence so that individuals become expert patients. They allow individuals to have more control over managing symptoms. These tools also promote healthy behaviors for a better quality of life.

PAHO has tools already available to support self-management of care: the *Passport to Healthy Lifestyle* (12) and the *Chronic Care Passport* (13). These tools help patients track variables, such as nutrition and physical activity, while teaching them to better manage their illness(es).

Evidence-based self-care programs, such as *Living a Healthy Life with Chronic Conditions* from Stanford University, have been implemented in 15 countries in the Americas in conjunction with PAHO. Also, more than 200 community leader teams are in place throughout the region (2). Impact evaluations have been carried out in five countries, demonstrating reduced emergency room visits (5%) at 6 and 12 months, and reduced hospitalizations (3%) at 6 months. Further evaluations are underway.

A new health care system must also consider adapting the environment within and beyond health services to optimize the functional ability of the elderly and disabled, allowing them to retain autonomy.

### Optimizing health outcomes

Priorities for designing a better system should focus on guaranteeing equitable access to and coverage by health services, strengthening intersectoral coordination, and ensuring optimal financing and efficiency (15). The quality of health care spending is critical to ensuring a sustainable health system, addressing inefficiencies, duplication, and unnecessary use of health services, testing, and treatments—currently estimated to waste as much as 40% of total expenditure by health care systems (16).

There is strong evidence to support the use of policy and legislation to reduce risk for NCDs and improve its management (17). The majority of NCD cases can be prevented and controlled with a series of cost-effective interventions outlined for NCD programs. Considering the high prevalence of risk factors within Aruba and Curaçao, affordable and evidence-based interventions would allow governments to use policy to reduce risk factors and improve care for those living with NCDs.

## WORKSHOP CONCLUSION

The demographic shift towards a larger elderly population, with its increased risks for developing NCDs and growing health care costs, jeopardizes the affordability and sustainability of health systems in Aruba and Curaçao. To address the situation, health authorities are rethinking the model of care and advocating for stronger primary health care, increased self-management, and treatment follow-up to prevent complications. Accomplishment of these goals requires a significant commitment to political engagement by the government and an intersectoral approach that comprises the social determinants of health.

## Disclaimer.

Authors hold sole responsibility for the views expressed in the manuscript, which may not necessarily reflect the opinion or policy of the *RPSP/PAJPH* and/or PAHO.
